# Pairwise Engineering of Tandemly Aligned Self-Splicing Group I Introns for Analysis and Control of Their Alternative Splicing

**DOI:** 10.3390/biom13040654

**Published:** 2023-04-06

**Authors:** Tomoki Ueda, Kei-ichiro Nishimura, Yuka Nishiyama, Yuto Tominaga, Katsushi Miyazaki, Hiroyuki Furuta, Shigeyoshi Matsumura, Yoshiya Ikawa

**Affiliations:** 1 Department of Chemistry, Graduate School of Science and Engineering, University of Toyama, Gofuku 3190, Toyama 930-8555, Japan; 2 Department of Chemistry and Biochemistry, Graduate School of Engineering, Kyushu University, Moto-oka 744, Nishi-ku, Fukuoka 819-0395, Japan; 3 Graduate School of Innovative Life Science, University of Toyama, Gofuku 3190, Toyama 930-8555, Japan

**Keywords:** alternative splicing, exon-inclusion, exon-skipping, group I intron, ribozyme, self-splicing, Tetrahymena

## Abstract

Alternative splicing is an important mechanism in the process of eukaryotic nuclear mRNA precursors producing multiple protein products from a single gene. Although group I self-splicing introns usually perform regular splicing, limited examples of alternative splicing have also been reported. The exon-skipping type of splicing has been observed in genes containing two group I introns. To characterize splicing patterns (exon-skipping/exon-inclusion) of tandemly aligned group I introns, we constructed a reporter gene containing two *Tetrahymena* introns flanking a short exon. To control splicing patterns, we engineered the two introns in a pairwise manner to design pairs of introns that selectively perform either exon-skipping or exon-inclusion splicing. Through pairwise engineering and biochemical characterization, the structural elements important for the induction of exon-skipping splicing were elucidated.

## 1. Introduction

**Figure 1:**
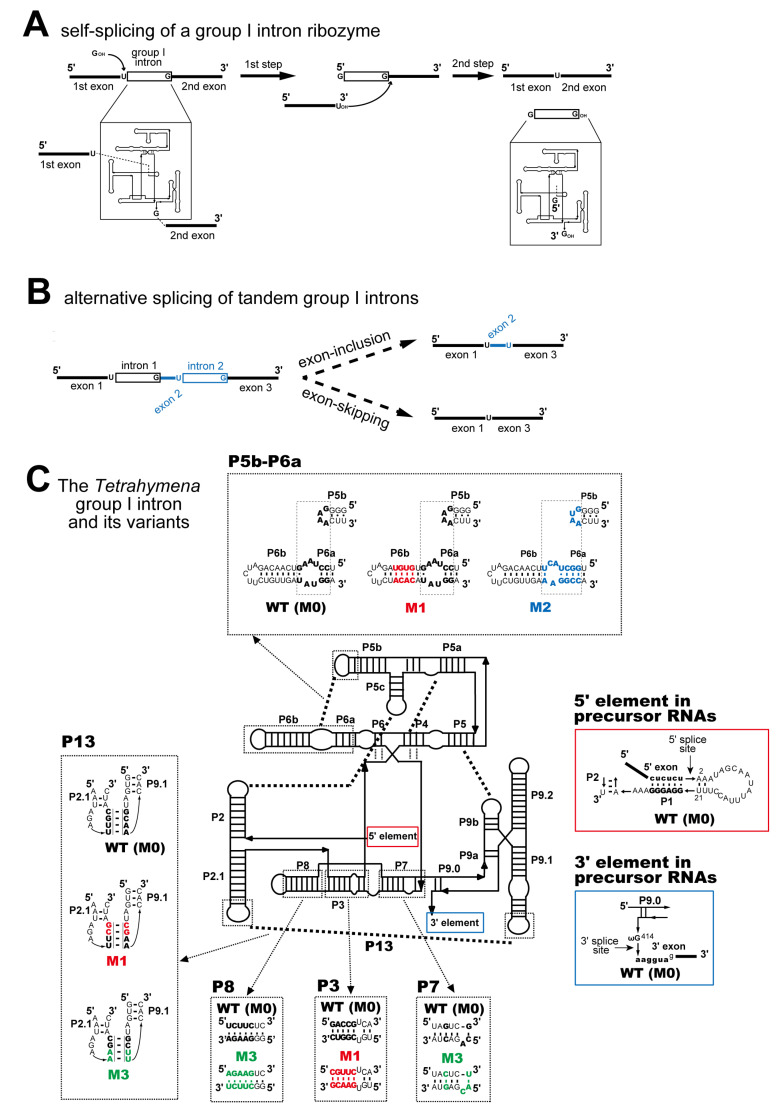
Self-splicing, alternative splicing, and secondary structures of group I intron ribozymes. (**A**) Schematic representation of self-splicing of a single group I intron ribozyme. The 5′ exon (1st exon) and 3′ exon (2nd exon) are shown as black lines. A group I intron ribozyme is shown as a thick white line. The last nucleotides of the 1st exon (U) and the intron (G) are shown. Schematic secondary structures of group I introns in the precursor form (left) and the excised form (right) are shown in boxes with solid lines. (**B**) Schematic representation of alternative splicing of two group I introns aligned in tandem with a short exon (exon 2). Exon 2 was present in exon-inclusion splicing and absent in exon-skipping splicing. (**C**) Secondary structures of the WT *Tetrahymena* group I ribozyme (WT or M0) and its variants (M1, M2, and M3). Colored nucleotides (red, blue, and green) in dotted boxes indicate the sequences of M1, M2, and M3, respectively. The 5′ and 3′ elements of the WT intron ribozyme are shown in red and blue boxes, respectively. Three thick broken lines indicate tertiary base pair interactions (P13 and P14) and a loop–receptor interaction (P5b–P6a). P13 and P5b–P6a interactions were modified to generate M1, M2, and M3 variants.

RNA splicing is a major class of processing events of primary transcripts synthesized via transcription of the corresponding DNA sequences. Primary transcripts commonly contain introns that must be removed in order to preserve their coding functions as messenger RNAs (mRNAs) or noncoding RNAs. In RNA splicing, introns are removed from the primary transcripts and the remaining RNA sequences (called exons) are joined together to yield mature transcripts. Introns in primary transcripts of eukaryotic nuclear protein-coding genes are recognized and removed by a large ribonucleoprotein complex called the spliceosome, in which RNA components are responsible for its catalytic function [1,2,3]. In addition to introns in pre-mRNAs removed by the spliceosome (spliceosomal introns), two other classes of introns that can remove themselves in an autocatalytic manner without *trans*-acting enzymes were identified [4,5,6,7]. This type of splicing is called self-splicing, and it is performed by the enzyme-like ability of intron RNA sequences (self-splicing introns). Self-splicing introns are classified into two groups designated as group I and group II introns [4,5,6,7]. Although their structures are quite different, the catalytic domain and splicing mechanism of group II introns share similarities with those of the spliceosome [8]. The spliceosome and group II introns have been proposed to have evolved from a common ancestral self-splicing intron resembling current group II introns [8]. Group I introns are considered to have evolved independently from group II introns and the spliceosome. The structure and function of group I introns have been studied extensively as the *Tetrahymena thermophila* group I intron (*Tetrahymena* intron, Figure 1) was the first RNA enzyme to be discovered [9,10], and it was shown to exhibit an excellent catalytic ability supported by its complex tertiary structure [11,12].

In splicing catalyzed by the spliceosome, primary transcripts bearing two or more introns often produce two or more different mature RNAs, in which particular exons of a given gene are either included within or excluded from the final RNA product. This type of splicing is called alternative splicing, which allows different mRNAs and proteins to be produced from a single gene [13,14]. Although the number of examples is limited, the alternative splicing of self-splicing group I and II introns has also been reported [15,16,17,18,19,20,21,22,23,24,25]. In genes containing a single group I intron sequence, distinct ligated exons can be produced by using alternative (or cryptic) 5′ or 3′ splice sites [19,20,21,22,23,24].

In genes containing two or more group I intron sequences, the removal of an exon flanked by two introns, which is known as exon-skipping splicing, has also been reported [25,26]. While exon-skipping splicing is frequently observed in spliceosomal splicing, it is quite rare in group I introns. Exon-skipping splicing was observed in the Orf142 gene in the *Staphylococcus aureus* bacteriophage Twort. Orf142 contains three group I introns, two of which (intron 2 and intron 3) are highly similar and belong to subgroup IA2 [25]. In self-splicing of intron 2 and intron 3, a short exon (23 nt) flanked by intron 2 and intron 3 is removed or retained as a product of either exon-skipping or exon-inclusion alternative splicing, respectively [24]. In the 23S rRNA gene containing two group I introns in *Coxiella burnetii*, exon-skipping splicing was observed with a short exon (34 nt) flanked by two group I introns belonging to different subgroups (IB2 and IA3) [26]. Exon-skipping splicing of two group I introns is interesting from a mechanistic viewpoint because the cooperative action of the two group I introns may be needed for their removal along with the exon between them. However, the occurrence of exon-skipping splicing was confirmed by RT-PCR analysis of the ligated exons [25,26], and no mechanistic analysis has been reported to date.

The cooperative splicing reactions of two group I intron ribozymes have also attracted attention in RNA-based synthetic biology because they may be applicable to the regulation of gene expression and gene therapy [27,28,29]. In these studies, structural and/or sequence engineering of the *Tetrahymena* intron ribozyme was performed to install cooperative functions between two monomeric introns. We applied this strategy to design and construct a model system with which mechanisms of exon-skipping splicing involving two group I introns could be investigated. We artificially designed precursor RNAs bearing two *Tetrahymena* introns separated by a short exon (24 nt). First, the splicing pattern of the artificial precursor RNA in *Escherichia coli* was characterized. To differentiate between the two introns, we then engineered the primary sequence of the *Tetrahymena* intron without sacrificing its tertiary structure [28]. Pairwise engineering of two intron ribozymes allowed us to construct precursor RNAs that predominantly perform either exon-skipping or exon-inclusion alternative splicing. Through sequence engineering and biochemical characterization, we elucidated the structural elements important for the induction of exon-skipping splicing.

## 2. Materials and Methods

### 2.1. Plasmid Construction

Plasmids possessing engineered *Tetrahymena* introns singly inserted into lacZ α-fragments were derived from pTZIVSU [30] and described previously [28]. Construction of a plasmid possessing the WT *Tetrahymena* introns in tandem with a short exon in the lacZ α-fragment was performed according to the following procedure: A pair of DNA fragments were prepared by PCR with pTZIVSU as a template. One DNA fragment consisted of the 5′ exon (exon 1), the WT *Tetrahymena* intron, and a 5′ portion of the short exon (exon 2) with a *Bsa*I site. The other DNA fragment consisted of a 3′ portion of exon 2 with a *Bsa*I site, the full-length intron, and the 3′ exon (exon 3). Each DNA fragment was digested with *Bsa*I, and the digested DNAs were then ligated to yield a fragment containing two introns flanking exon 2. The resulting fragment was then digested with *Eco*RI and *Hin*dIII and inserted into the pTZ18U vector digested with *Eco*RI and *Hin*dIII. Each plasmid possessing a given pair of engineered introns was prepared in a similar manner by using a given pair of DNA fragments, each of which was amplified by PCR from a plasmid bearing the corresponding engineered intron.

### 2.2. β-Galactosidase Assay

Each plasmid containing a single intron or a pair of introns flanking exon 2 was used to evaluate β-galactosidase activity. β-Galactosidase assay with TokyoGreen-β-d-galactoside (TokyoGreen β-gal; Goryo Chemical, Sapporo, Japan) was performed according to the following procedure: Plasmid pTZIVSU and its derivatives without or containing one or two introns were transformed into *E. coli* JM109. Individual colonies bearing plasmids were inoculated into 3 mL of lysogeny broth (LB) containing 100 μg/mL of ampicillin and were incubated overnight at 30 °C with shaking at 210 rpm. Aliquots of 40 μL of individual cell cultures were added to 4 mL of fresh LB containing 100 μg/mL of ampicillin. The cultures were incubated at 30 °C with shaking at 210 rpm until OD_600_ increased to about 0.55. Then, 4 μL of 1.0 M IPTG (final concentration 1.0 mM) was added to the cultures and further incubated at 37 °C with shaking at 180 rpm for 14 h. Samples of 1 mL of each cell culture were centrifuged at 3000× *g* for 5 min and the supernatants were discarded. The resulting cell pellets were washed with a mixture of LB broth and MOPS buffer (100 mM MOPS, 100 NaCl, pH 8.0) four times, which caused the ratio of LB broth to MOPS buffer to change gradually from 8:2 to 1:9. The cell pellets were then suspended in MOPS buffer containing 50% Percoll, and OD_600_ was adjusted to 1.0. The resulting cell suspensions (10 μL) were mixed with an equal volume (10 μL) of permeabilization solution containing 100 mM MOPS, 100 mM NaCl, 35% BugBuster Protein Extraction Reagent (Merck, Darmstadt, Germany), and 2.0 μM TokyoGreen β-gal. The mixtures were added to microtiter plates, which were then immediately placed in a prewarmed plate reader (Infinite F200 Pro; Tecan, Männedorf, Switzerland) and incubated at 37 °C. Fluorescence intensity was monitored every 1 min. The β-galactosidase activity was evaluated from the early phase of the enzyme reaction, during which the fluorescence from the product increased linearly.

### 2.3. Reverse Transcription-PCR (RT-PCR)

Plasmid pTZIVSU and its derivatives without or containing one or two introns were transformed into *E. coli* JM109. Individual colonies were grown overnight at 30 °C with shaking at 210 rpm in LB medium containing ampicillin (100 μg/mL). The procedure and culture conditions were the same as described above to express lacZ α-fragment mRNA precursors containing introns to evaluate β-galactosidase activity. After the induction of lacZ α-fragment mRNA precursors through the addition of IPTG and further incubation at 37 °C for 14 h, the total RNA was extracted from the resulting *E. coli* cells in the cultures using RNAprotect Bacteria Reagent and RNeasy Mini Kit (Qiagen, Hilden, Germany) according to the manufacturer’s instructions. Total RNAs were then treated with RQ1 RNase-free DNase I (Promega, Madison, WI, USA) to remove residual genomic DNA. Reverse transcription was performed using ReverTra Ace (Toyobo, Kyoto, Japan) and a primer (5′-CTGGCGAAAGGGGGATG-3′). The resulting cDNA was then used as a template for PCR with a set of primers (5′-GAATTCGAGCTCGGGTAAC-3′ and 5′-CTGGCGAAAGGGGGATG-3′) to amplify a short DNA fragment across the splice junctions of exon-skipping and exon-inclusion splicing products. The length of the PCR products depended on the presence (174 nt) or absence (150 nt) of exon 2.

## 3. Results

### 3.1. In Vivo Splicing of Precursor RNAs Containing Tetrahymena Introns in Tandem

**Figure 2:**
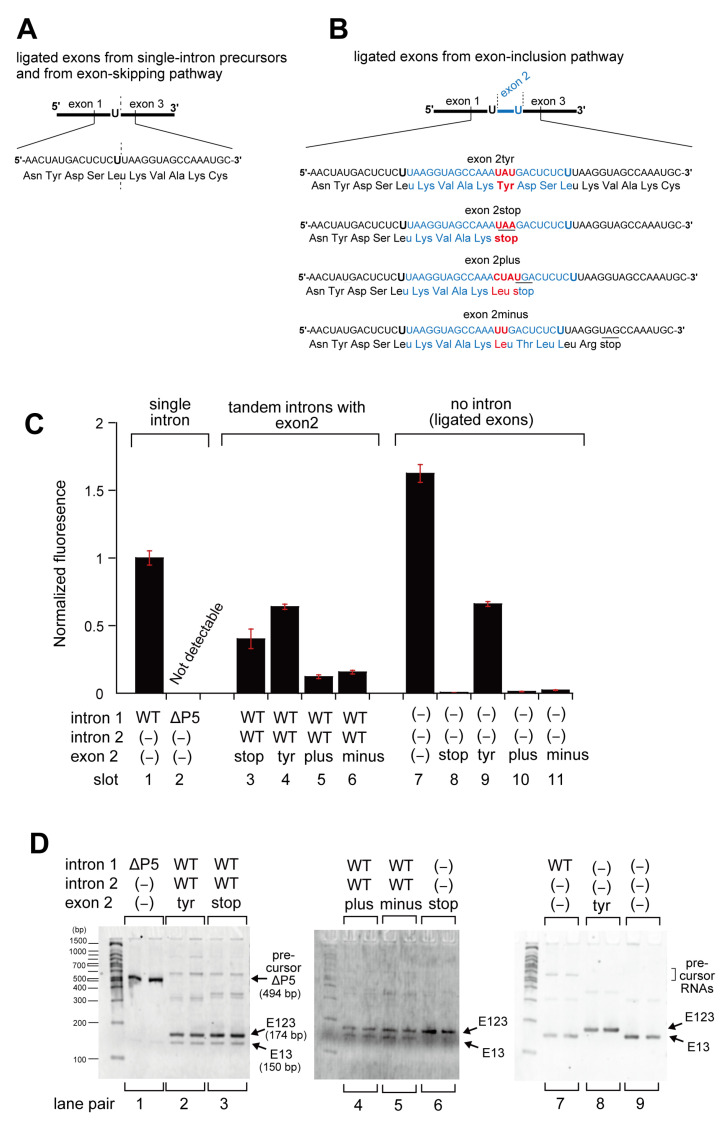
Splicing patterns of precursor RNAs possessing two *Tetrahymena* introns. (**A**) Ligated exons from single-intron precursors and exon-skipping splicing. The corresponding amino acid sequence is also shown. (**B**) Ligated exons from exon-inclusion splicing. Four exon 2 sequences were used in this study. The corresponding amino acid sequences are also shown. (**C**) β-Galactosidase reporter gene assays to evaluate the splicing activities of the precursors with a single WT or ∆P5 intron and four precursors with two introns and a short exon (exon 2) in *E. coli*. Expression of the lacZ-α fragment was also measured from the ligated exons without or with exon 2. (**D**) RT-PCR assays to distinguish the ligated exons (E123 or E13) including or skipping exon 2, with lengths of 174 and 150 bp, respectively. As the ∆P5 mutant was splicing-deficient in *E. coli*, the RT-PCR product corresponded to the precursor RNA.

We designed and constructed a model system containing two identical group I introns flanking a short exon (Figure 1B). Two *Tetrahymena* group I intron ribozymes were inserted flanking a short exon into the lacZ α-fragment gene because a derivative of the lacZ α-fragment gene has been developed by inserting a single *Tetrahymena* intron at the same position and used to evaluate self-splicing of the single-intron ribozyme in *E. coli* (Figure 1A and Figure 2A) [30]. In the presence of the lacZ ω-fragment provided by the host *E. coli*, the extent of self-splicing was reflected in the production of a functional β-galactosidase α-fragment translated from the ligated exons (Figure 2A). In this study, we monitored the amount of functional β-galactosidase using the fluorescent reporter substrate TokyoGreen-βGal [31].

We first evaluated the splicing activities of the wild-type (WT) *Tetrahymena* intron ribozyme and its ∆P5 mutant as positive and negative controls, respectively. The extent of their splicing in *E. coli* was determined by monitoring the change in fluorescence with the hydrolysis of TokyoGreen-βGal treated with *E. coli* cell lysate [32]. In vitro splicing experiments in the presence of a low concentration (<5 mM) of Mg^2+^ showed that the WT intron ribozyme underwent efficient self-splicing, whereas the ∆P5 mutant ribozyme was nearly inactive [33]. Treatment of TokyoGreen-βGal with cell lysate containing the WT intron ribozyme exhibited fluorescence (slot 1 in Figure 2C), while the same assay using the lysate containing the ∆P5 intron ribozyme exhibited no detectable fluorescence (slot 2 in Figure 2C). These results were consistent with a previous study in which a similar assay was performed using X-gal as a reporter substrate [28]. To further evaluate the splicing ability of the precursor RNAs with WT and ∆P5 introns, the length of their lacZ mRNAs was compared by RT-PCR amplification (Figure 2D). RNA samples isolated from *E. coli* cells with the WT intron exhibited a recognizable band (150 bp) corresponding to the ligated exons (E13), whereas the band of 563 bp that corresponded to the RT-PCR product of the precursor RNA was very weak (lane pair 7 in Figure 2D). RNA samples isolated from *E. coli* cells with the ∆P5 intron yielded a major PCR product of 494 bp corresponding to the precursor RNA, whereas no PCR products were observed corresponding to the ligated exons (150 bp) (lane pair 1 in Figure 2D).

We then examined the splicing reaction of the precursor lacZ α-fragment RNA containing two *Tetrahymena* ribozymes arranged in tandem and flanking a short (24 nt) exon. We designed the short exon sequence to provide the cognate 3′ splice site of the first intron (intron 1) as well as the cognate 5′ splice site of the second intron (intron 2) (Figure 2B). We installed an in-frame stop (TAA) codon in the expected product of the exon-inclusion reaction (Figure 1B) to detect the inclusion of the short exon (exon 2stop) as a deficiency of lacZ activity (Figure 2B). Regarding exon 2stop, a functional lacZ α-fragment can be produced only through exon-skipping splicing (Figure 1B). We also prepared a variant of exon 2 (exon 2tyr), the sequence of which was identical to exon 2stop except for a single A to U base change to alter the stop (TAA) codon to a tyrosine (TAT) codon (Figure 2B). With exon 2tyr, a functional lacZ α-fragment can be produced regardless of the splicing pattern. We also prepared two exon 2 variants in which a single nucleotide was added to or removed from exon 2tyr (exon 2plus and exon 2minus, respectively). The resulting two variants were expected to produce nonfunctional mRNAs in exon-inclusion splicing (Figure 2B).

We prepared plasmids carrying the sequences of splicing products (ligated exons) produced by single-intron splicing and exon-skipping splicing. We also prepared four plasmids bearing sequences of exon-inclusion splicing with four exon 2 sequences (stop, tyr, plus, and minus). The splicing reactions of these constructs in *E. coli* were evaluated by adding TokyoGreen-βGal to their cell lysates. The cell lysate with the ligated exons (i.e., the exon-skipping product) exhibited 1.6-fold higher fluorescence intensity than cell lysate with a splicing-competent precursor RNA bearing a single intron (slots 1 and 7 in Figure 2C). The cell lysate with the ligated exons bearing exon 2tyr (i.e., the exon-inclusion product) also exhibited fluorescence, although, its intensity was 0.7-fold that with the splicing-competent precursor RNA bearing a single WT intron (slots 1 and 9 in Figure 2C). Three ligated exons designed to produce nonfunctional lacZ α-fragment mRNAs showed no (exon 2stop) or nearly no (exon 2plus and exon 2ninus) fluorescence intensity (slots 8, 10, and 11 in Figure 2C).

Based on these experimental results, we then analyzed the in vivo splicing of precursor RNAs containing two WT *Tetrahymena* intron ribozymes and a short exon 2 (slots 3–6 in Figure 2C). In the TolyoGreen-βGal assay, *E. coli* lysate containing exon 2tyr exhibited fluorescence intensity that was 0.7-fold that with the single WT ribozyme (slots 1 and 4 in Figure 2C). Interestingly, the replacement of exon 2tyr with exon 2stop resulted in only a moderate decrease in fluorescence intensity (slots 3 and 4 in Figure 2C). While this result suggested exon-skipping splicing as the major splicing pattern, the moderate decrease in fluorescence intensity also suggested that exon-inclusion splicing contributed to the production of active lacZ α-fragment in the cell lysate with exon 2tyr. Additional support for exon-skipping splicing was also provided by the moderate fluorescence intensity in cell lysates containing exon 2plus and exon 2minus (slots 5 and 6 in Figure 2C) because their exon-inclusion splicing products were unable to produce a functional lacZ α-fragment. RT-PCR analyses also confirmed the occurrence of exon-skipping and exon-inclusion splicing in their precursor RNAs (Figure 2D and Figure S2). Four RNA samples prepared from *E. coli* cells exhibited two bands on RT-PCR with lengths corresponding to the ligated exons with and without exon 2 (E123 and E13), respectively, (lane pairs 2–4 in Figure 2D, see also Figure S3A). We isolated 150 bp and 174 bp RT-PCR products and cloned them into T-vector pMD19 (Takara Bio, Shiga, Japan). We determined their sequences and confirmed that 150 bp and 174 bp RT-PCR products actually had the predicted sequences produced by exon-skipping and exon-inclusion splicing, respectively.

### 3.2. Pairwise Engineering of Tandem Introns to Suppress Exon-Inclusion Splicing

**Figure 3:**
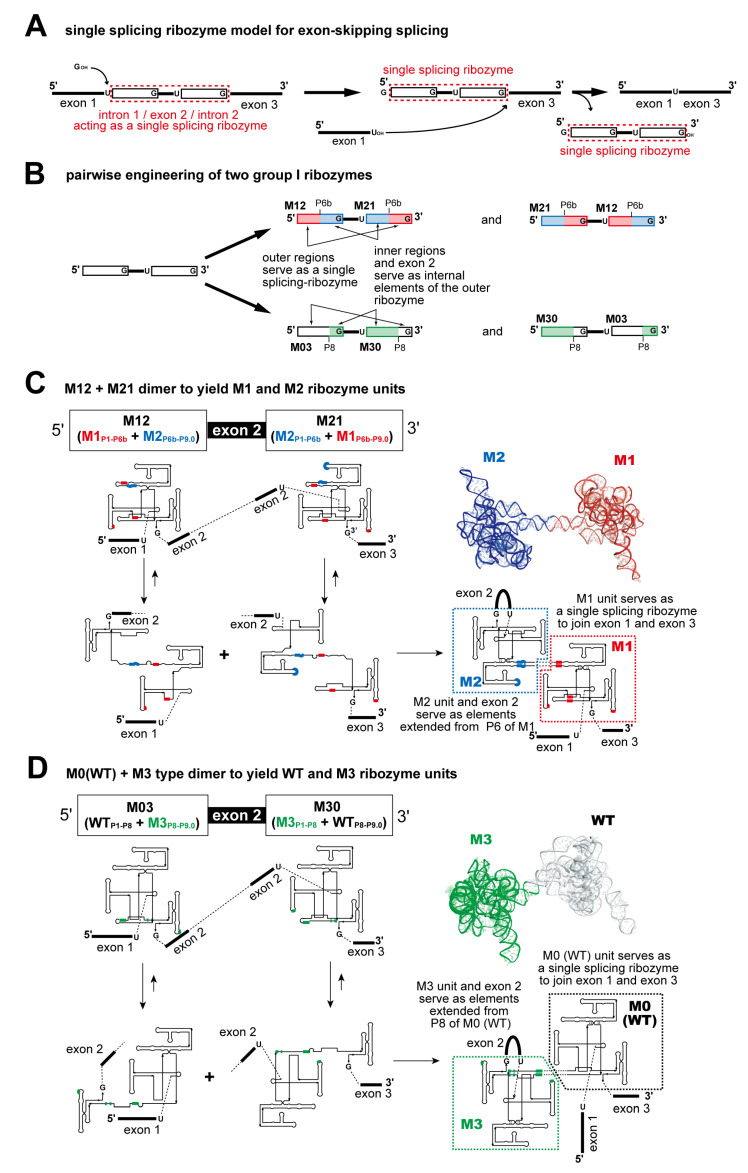
Rational engineering of tandemly aligned introns to induce exon-skipping splicing. (**A**) Schematic representation of a single splicing ribozyme model for exon-skipping splicing. Intron 1, exon 2, and intron 2 enclosed with red broken lines act as a single splicing ribozyme. (**B**) Pairwise engineering of two group I introns to eliminate their splicing activities. Two introns were engineered to form an active ribozyme to cooperatively join exon 1 and exon 3. (**C**) Secondary and 3D structures of a pair of chimeric introns (M12  +  M21). In 3D structures, active ribozymes organized by the pairing of two chimeric introns are shown in blue (M1) and red (M2). (**D**) Secondary and 3D structures of a pair of chimeric introns (M03  +  M30). In 3D structures, active ribozymes organized by the pairing of two chimeric introns are shown in green (M3) and gray (WT).

As precursor RNAs containing two WT *Tetrahymena* introns flanking the short exon (exon 2) conducted both exon-skipping and exon-inclusion splicing in *E. coli*, we then designed pairs of ribozymes capable of performing one of the two splicing patterns exclusively. For this purpose, we used engineered *Tetrahymena* ribozymes (M1, M2, and M3), the primary sequences of which were engineered without sacrificing the tertiary structure and catalytic activity of the parent WT ribozyme (referred to hereafter as M0) (Figure 1C and Figure S1) [28]. We first designed two pairs of ribozyme variants that would induce exon-skipping splicing exclusively. According to the single ribozyme model shown in Figure 3A, we performed the molecular engineering of two ribozymes in a pairwise manner (Figure 3B and Figure S3A). 

**Figure 4:**
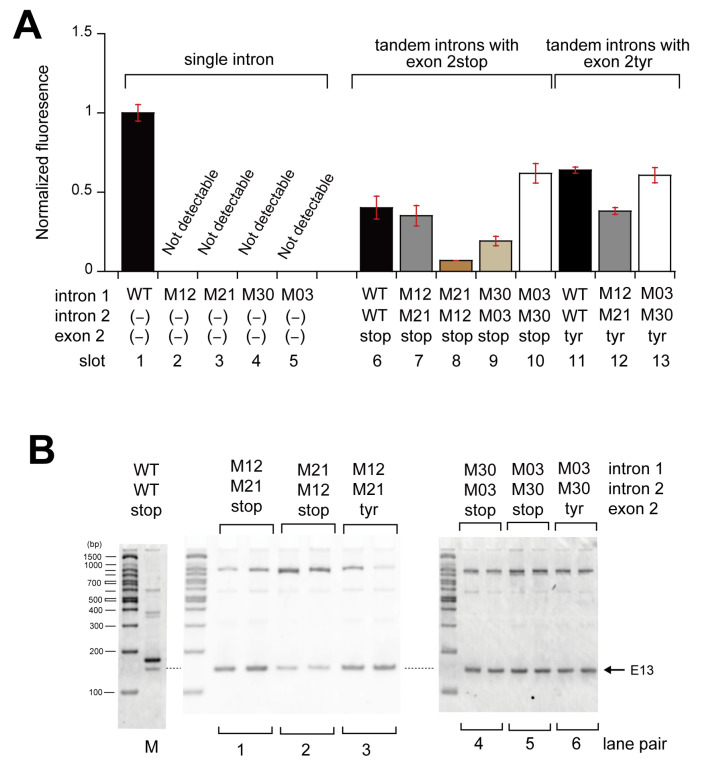
Exon-skipping splicing conducted by pairs of chimeric group I introns. (**A**) β-Galactosidase reporter gene assays to evaluate the splicing activities of precursors with WT, ∆P5, and four chimeric introns as well as eight precursors with two introns and a short exon (exon 2) in *E. coli*. (**B**) RT-PCR assays to distinguish the ligated exons (E123 or E13) including or skipping exon 2, with lengths of 174 and 150 bp, respectively.

We divided the sequences of M1 and M2 variants at the terminal loops of their P6b elements. The resulting upstream and downstream sequences were swapped to generate two chimeric variants, M12 (M1_P1–P6b_ + M2_P6b–P9.0_) and M21 (M1_P1–P6b_ + M2_P6b–P9.0_), which served as the first ribozyme pair (Figure 3B,C). We also divided the WT ribozyme and M3 variant at their P8 element and swapped their upstream and downstream sequences. The resulting chimeric variants of M03 (WT_P1–P8_ + M3_P8–P9.0_) and M30 (M3_P1–P8_ + WT_P8–P9.0_) were used as the second pair of ribozymes (Figure 3B,D) [28]. In a pair of M12 and M21 variants, the P1–P6b region of the M12 (or M21) ribozyme would be assembled with the P6b–P9.0 region of the M21 (or M12) ribozyme to form the active structure of the M1 (or M2) ribozyme in a pseudo-bimolecular format (Figure 3C). In a pair of M03 and M30 variants, the P1–P8 region of the M03 (or M30) ribozyme would be assembled with the P8–P9.0 region of the M30 (or M03) ribozyme to form the active structure of the WT (or M3) ribozyme in a pseudo-bimolecular format (Figure 3D). In the TokyoGreen-βGal assay, cell lysates containing the four chimeric variants showed no detectable fluorescence suggesting a significant disturbance of their self-splicing activities in *E. coli* (slots 2–5 in Figure 4A). In each of the four chimeric variants, a catalytically active structure could not be established due to the disruption of some elements in the conserved core and some tertiary interactions supporting the core element (Figure 3C,D). In the precursor RNAs containing two intron ribozymes, defects in the self-splicing ability of each intron would eliminate exon-inclusion splicing because this splicing pattern corresponds to two independent splicing events of two intron ribozymes.

We used two pairs of ribozymes (M12/M21 pair and M03/M30 pair) as two introns inserted into the precursor RNAs. We first prepared four DNA constructs to produce four precursor RNAs with exon 2stop. Two contained the M12 and M21 variants, with the remaining two containing M03 and M30 (Figure 3B). In the TokyoGreen-βGal assay, cell lysates with four precursor RNAs all exhibited fluorescence, although the intensities varied among the four precursors (slots 7–10 in Figure 4A). These observations indicated that precursor RNAs containing the M12 and M21 pair and those containing the M03 and M30 pair can perform exon-skipping splicing in *E. coli*. The presence of exon 2 in the ligated exons was analyzed by RT-PCR. No PCR product corresponding to exon-inclusion splicing was observed in the four RNA samples (lane pairs 1, 2, 4, and 5 in Figure 4B, see also Figures S3A and S4). This result strongly suggested that the four precursor RNAs performed exon-skipping splicing exclusively in *E. coli* cells.

To further examine the occurrence of exon-inclusion splicing of these four variants, we replaced exon 2stop of M12 and M03 variants with exon 2tyr. If exon-inclusion splicing also proceeded in these variants in parallel with exon-skipping, the replacement of their exon 2stop with exon 2tyr would increase the amount of the functional lacZ α-fragment and enzymatic activity. In the TokyoGreen-βGal assay, cell lysates with the variants of M12 and M03 bearing exon 2tyr exhibited no increase in their reporter fluorescence compared to those bearing exon 2stop (slots 7, 10, 12, and 13 in Figure 4A). This observation further supported the suppression of exon-inclusion splicing in the precursor RNAs possessing a pair of chimeric-intron ribozymes and was also consistent with the results of the RT-PCR analysis (lane pairs 3 and 6 in Figure 4B).

### 3.3. Pairwise Engineering of a Tandem Intron Ribozyme to Suppress Exon-Skipping Splicing

**Figure 5:**
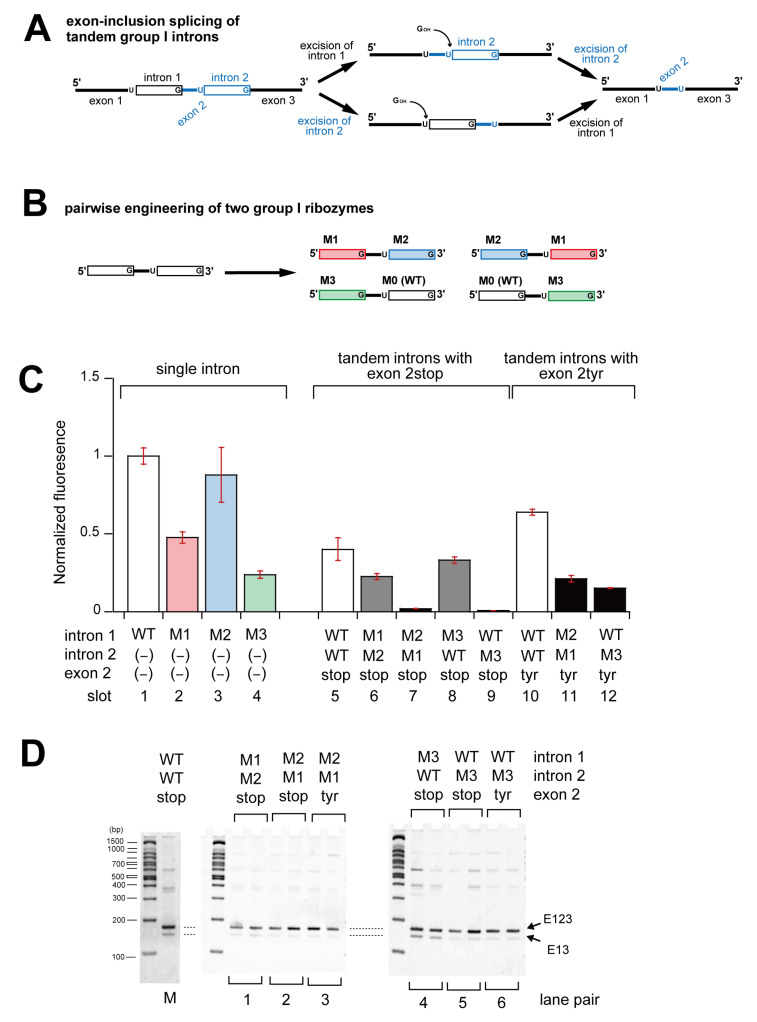
Rational engineering of tandemly aligned introns to induce exon-inclusion splicing. (**A**) Schematic representation of exon-inclusion splicing. Intron 1 and intron 2 carry out self-splicing independently. (**B**) Pairwise engineering of two group I introns to retain the splicing activity of each intron but eliminate the formation of a single active ribozyme cooperatively from intron 1 and intron 2. (**C**) β-Galactosidase reporter gene assays to evaluate the splicing activities of four precursors with WT as well as three variants and eight precursors with two introns and a short exon (exon 2) in *E. coli*. (**D**) RT-PCR assays to distinguish the ligated exons (E123 or E13) including or skipping exon 2, with lengths of 174 and 150 bp, respectively.

We investigated exon-inclusion splicing as a result of the independent splicing of tandemly aligned ribozymes (Figure 5A). To prevent the formation of a single ribozyme structure through interaction between the 5′ portion of the first ribozyme and the 3′ portion of the second ribozyme, we differentiated the two ribozymes flanking exon 2. We used a pair of engineered variants (M1 and M2), a pair consisting of WT (i.e., M0), and a variant (M3), from which four precursor RNAs were designed and prepared (Figure 5B). Preliminary assays with three engineered introns (M1, M2, and M3) exhibited their splicing ability in *E. coli* (slots 2–4 in Figure 5C). This was consistent with the results of previous experiments using X-gal as a substrate. To detect the occurrence of exon-skipping splicing, we first used exon 2stop as the short exon. In the TokyoGreen-βGal assay, the lack or poor occurrence of exon-skipping splicing would result in no or nearly no production of fluorescence.

Cell lysate containing a precursor RNA with M2 and M1 as the first and second introns (M2–M1) exhibited nearly no fluorescence (slot 7 in Figure 5C). A similar result was also obtained with the cell lysate containing a precursor RNA with WT and M3 as the first and second introns (M0–M3) (slot 9 in Figure 5C). To exclude the possibility of a simple splicing defect in the respective introns, we replaced exon 2stop with exon 2tyr. Cell lysates containing precursor RNAs of M2–M1 and M0–M3 exhibited fluorescence when they possessed exon 2tyr (slots 11 and 12 in Figure 5C), thus indicating that these precursors produced a functional lacZ α-fragment through exon-inclusion splicing. RT-PCR analysis of mRNAs of M2–M1 and M0–M3 variants showed predominant production of E123, whereas they also produced detectable amounts of PCR products corresponding to E13 (lane pairs 2, 3, 5, and 6 in Figure 5D). In M2–M1 and M0–M3 constructs, the ratios of E13 to E123 PCR products were markedly smaller than in WT–WT constructs (Figures S3A and S5). These results strongly suggested that M2–M1 and M0–M3 precursor RNAs perform exon-inclusion splicing nearly exclusively, yielding E123.

While precursor RNAs of M2–M1 and M0–M3 appeared to perform exon-inclusion splicing exclusively, the remaining two precursor RNAs (M1–M2 and M3–M0) exhibited fluorescence despite possessing exon2stop as the second intron. This observation indicated that the two precursor RNAs of M1–M2 and M3–M0 can perform exon-skipping splicing although they cannot form single ribozyme structures. It was, therefore, necessary to propose a model for exon-skipping splicing without the cooperative formation of single ribozyme structures (Figure 3A) but with the independent formation of two active ribozymes.

### 3.4. Engineering of Splice Site Recognition Elements as an Alternative Strategy to Suppress Exon-Skipping Splicing

**Figure 6:**
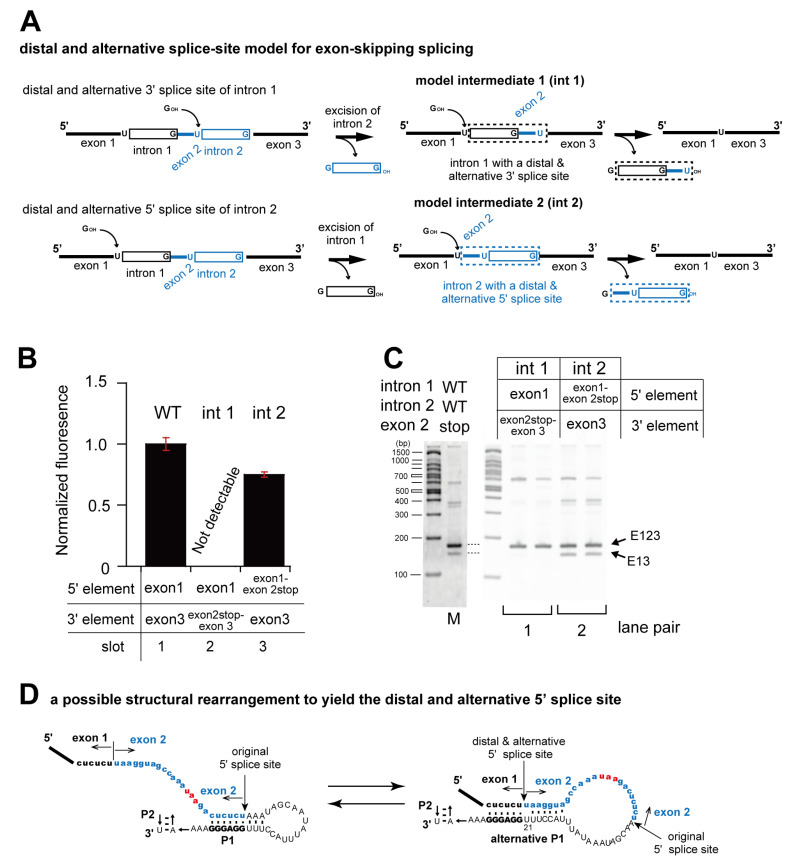
Distal and alternative splice sites induce exon-skipping splicing. (**A**) Schematic representations of distal and alternative splice-site models for exon-skipping splicing. (**B**) β-Galactosidase reporter gene assays of model intermediates to evaluate their use of distal and alternative splice sites to yield the ligated exons lacking exon 2 in *E. coli*. (**C**) RT-PCR assays to distinguish the ligated exons (E123 or E13) including or skipping exon 2, with lengths of 174 and 150 bp, respectively. (**D**) A possible structural rearrangement to yield the distal and alternative 5′ splice site.

We considered the use of alternate splice sites, which is an alternative splicing strategy for pre-mRNA catalyzed by the spliceosome. Although alternate (or cryptic) splice sites are not commonly used in self-splicing of group I introns, several examples of self-splicing or its related reactions at cryptic splice sites were reported in group I ribozymes. We hypothesized that there were intermediate RNAs in which only the second or first intron ribozyme completed the splicing reaction. In the intermediate RNAs retaining the first intron, the exon–exon junction of exon 2stop/exon 3 may serve as an alternate 3′ splice site (top of Figure 6A). In the intermediate RNAs retaining the second intron, the exon–exon junction of exon 1/exon 2stop may serve as an alternate 5′ splice site (bottom of Figure 6A) [19,20,21,22,23,24].

We prepared plasmids possessing the intermediate sequences and introduced them into *E. coli*. In the TokyoGreen-βGal assay, the cell lysate containing ligated exons (exon 2stop + exon 3) as a 3′ exon for intron 1 exhibited no detectable fluorescence (slot 2 in Figure 6B). On the other hand, the cell lysate containing ligated exons (exon 1 + exon 2stop) as a 5′ exon for intron 2 exhibited fluorescence (slot 3 in Figure 6B). In the RT-PCR analysis, no PCR product corresponding to exon-skipping splicing (E13) was detected in an isolated RNA from cell lysate containing ligated exons (exon 2stop + exon 3) as a 3′ exon for intron 1 (lane pair 1 in Figure 6C and Figure S6). On the other hand, a PCR product corresponding to exon-skipping splicing was observed in an isolated RNA from cell lysate containing ligated exons (exon 1 + exon 2stop) as a 5′ exon for intron 2 (lane pair 2 in Figure 6C, see also Figure 3A). Based on these observations, we proposed a structural model in which ligated exons (exon 1 + exon 2stop) functionally serve as a 5′ exon to form the distal and alternative P1 element capable of conducting both the first and second steps of the splicing reaction (Figure 6D). As one of the intermediate RNAs (int 2 RNA) can use the alternate splice site to produce the exon-skipping splicing product, we suppressed the formation of the alternate splice site(s) by engineering the structural elements defining the 5′ and 3′ splice sites.

**Figure 7:**
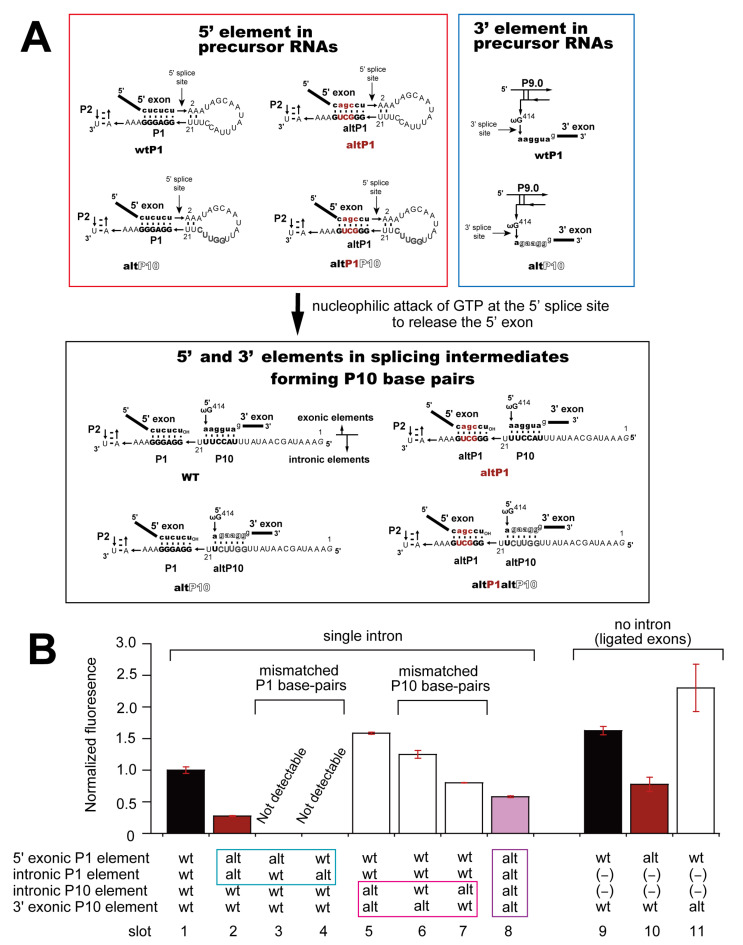
Rational engineering of the 5′ and 3′ splice sites to distinguish two introns. (**A**) The 5′ and 3′ elements of the WT intron ribozyme and its variants are shown in red and blue boxes, respectively. P10 base pairs formed in splicing intermediates by their 5′ and 3′ elements are shown in the black box. (**B**) β-Galactosidase reporter gene assays to evaluate the splicing activities of eight precursors with single introns possessing the WT and variant P1 and P10 elements. Expression of the lacZ-α fragment from the ligated exons yielded by variant P1 and P10 elements was also measured.

We engineered the P1 base pair element formed between exon 2 and intron 2 (Figure 7A). As the resulting P1 element in intron 2 was distinct from that of intron 1, the intronic P1 element of intron 2 did not recognize the ligated exons (exon 1 + exon 2stop) as the 5′ exonic P1 element. We then additionally modified the structural elements (P9.2, P9.0, and P10) participating in the definition and recognition of the 3′ splice site (Figure 7A). As the modification of P1 and P10 elements inevitably altered the exonic sequence encoding amino acids (Figure S7A), we performed a preliminary examination of the effects of these P1 and P10 modifications using a precursor RNA bearing a single intron (Figure 7B).

A variant intron with altered P1 elements (altP1) produced a functional lacZ α-fragment, although, fluorescence intensity was 0.28-fold that of the WT intron in the TokyoGreen-βGal assay (slots 1 and 2 in Figure 7B). The cell lysate containing the intron-less (ligated exons) construct of altP1 also exhibited lower fluorescence intensity than the lysate of *E. coli* carrying the product produced by the plasmid of the intron-less (ligated exons) construct WT P1 (slots 9 and 10 in Figure 7B). This observation suggested that the reduction of fluorescence intensity in the altP1 construct was at least partially due to the altered efficiency of translation and/or enzyme activity of the lacZ α-fragment. The importance of the P1 element was also confirmed by the two mutant precursors with disrupted P1 base pairs (Figure S7B) because the cell lysates containing them exhibited no fluorescence in the TokyoGreen-βGal assay (slots 3 and 4 in Figure 7B).

We also examined a variant precursor with an altered P10 element (Figure 7A), the fluorescence intensity of which was 1.6-fold higher than the parent intron in the TokyoGreen-βGal assay (slot 5 in Figure 7B). This difference was also due to the distinct translation and/or enzymatic efficiency of the lacZ α-fragment (slot 11 in Figure 7B). The disruption of P10 elements (Figure S7B) reduced but did not abolish the splicing activity (slots 6 and 7 in Figure 7B). The self-splicing activity of a variant with both altP1 and altP10 mutations was also examined, and its fluorescence intensity was 0.58-fold that of the WT intron (slot 8 in Figure 7B).

**Figure 8:**
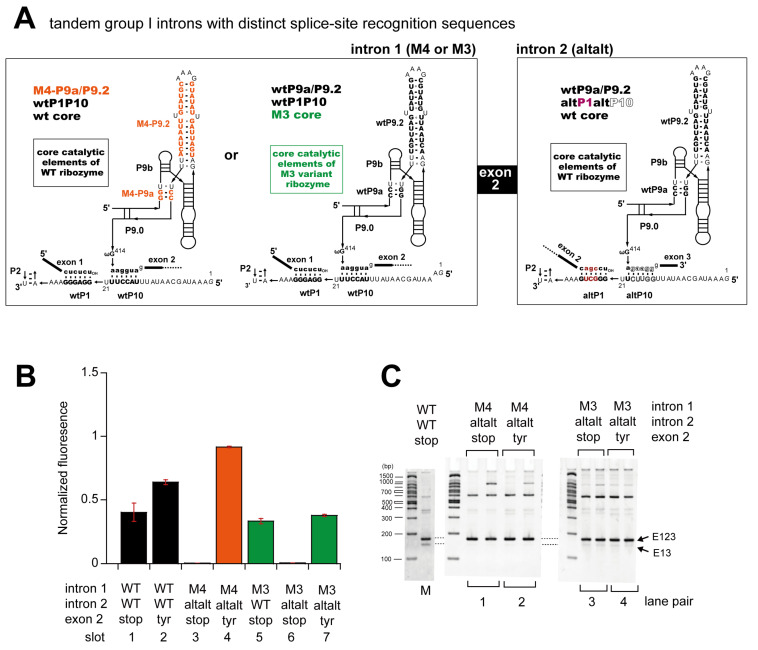
Suppression of exon-skipping splicing in tandemly aligned introns with distinct splice site recognition sequences. (**A**) P9a/9.2, P1, and P10 elements in tandem introns with distinct splice site recognition sequences. (**B**) β-Galactosidase reporter gene assays to evaluate the splicing activities of precursors with two introns and a short exon (exon 2) in *E. coli*. (**C**) RT-PCR assays to distinguish the ligated exons (E123 or E13) including or skipping exon 2, with lengths of 174 and 150 bp, respectively.

Based on the results for single intron constructs, we then constructed and examined tandem introns by combining the altP1P10 variant and the M4 or M3 intron variant. The M4 variant altered P9.2 and P9a elements but caused no alternations in the core elements of the intron ribozyme (Figure 8A) [28]. The ribozyme activity of the M4 variant was shown to be close to that of the WT intron in vitro and also in *E. coli*. In the tandem introns consisting of the M4 variant and the altP1altP10 variant, four elements involved in the splice site recognition (P1, P10, P9a, and P9.2) were all different between the first intron (M4) and second intron (altP1altP10) (Figure 8A). The cell lysate containing the tandem introns of the M4 variant and the altP1P10 variants (abbreviated as altalt in Figure 8B,C) flanking exon 2stop showed no fluorescence (slot 3 in Figure 8B), suggesting that the differentiation of the splice site recognition elements sufficiently suppressed exon-skipping splicing. The RT-PCR analysis also supported the exclusive occurrence of exon-inclusion splicing (lane pair 1 in Figure 8C and Figure S8). When we replaced exon 2stop with exon 2tyr, the cell lysate containing the tandem introns of the M4 variant and the altP1altP10 variants showed β-galactosidase activity in the TokyoGreen-βGal assay (slot 4 in Figure 8B) with no changes in the band pattern of the RT-PCR assay (lane pair 2 in Figure 8C). These results further supported the selective occurrence of exon-inclusion splicing. Exon-skipping splicing observed in M3–M0 (tandem introns of M3 and WT) was suppressed nearly completely through the replacement of the WT intron with the altP1altP10 variant (slot 6 in Figure 8B and lane pair 3 in Figure 8C), and the recovery of the β-galactosidase activity was also observed through the substitution of exon 2stop with exon 2tyr (slot 7 in Figure 8B) with no changes in the band pattern of the RT-PCR assay (lane pair 4 in Figure 8C). These results indicated that the differentiation of splice site recognition elements is an evolutionally effective approach to select the exclusive exon-inclusion splicing pattern.

## 4. Discussion

In this study, we engineered tandemly arranged *Tetrahymena* introns flanking a short exon in a pairwise manner to control alternative splice patterns. A predominant selection of the exon-skipping pattern could be achieved by the inactivation of every single intron without sacrificing the cooperative formation of a single large ribozyme by two introns. The formation of a single large ribozyme has been proposed as a possible mechanism of the exon-skipping splicing of two highly similar group IA2 introns [25]. On the other hand, the exon-inclusion pattern was not necessarily selected by the differentiation of two intron ribozymes through sequence engineering. The selective disruption of the cooperative formation of a single large ribozyme succeeded in conducting exon-inclusion splicing in some constructs. Some other constructs, however, still yielded an exon-skipping splicing product. We showed that the exon-skipping products in these constructs were formed through the stepwise splicing of two introns. The first intron self-spliced normally to yield an intermediate containing the short exon and the second intron. The second intron used the splice junction of the first intron as a distal and alternative splice site for the second intron ribozyme, the P1 element of which was reorganized to include the short exon (Figure 6D). A naturally occurring example of a distal and alternative 5′ splice site has been identified in the *Tetrahymena*-type (group IC1) intron in *Physarum* amoeba [20]. The *Physarum* group IC1 intron is highly similar to that of *Tetrahymena* and inserted at the exact same site in the nuclear rDNA [20]. This group IC1 intron, however, is mobile and encodes a homing endonuclease. The alternative splicing appeared to skip a short exon (63 nucleotides) in ligated RNA, and its biological significance may be linked to the expression of the intron protein and homing mobility [20].

The exon-skipping product through stepwise splicing observed in the construct with the second intron ribozyme (M2 or WT) was more active than the first intron (M1 or M3) (Figure 5). As the use of the distal and alternative 5′ splice site must be less efficient than the authentic splice site, the second intron must be sufficiently active to use the less efficient 5′ splice site. On the other hand, the efficiency of this type of exon-skipping mechanism would be limited, because the second intron would tend to self-splice faster than the less active first intron, and the resulting intermediate would be unable to use the splice junction of the second intron as the distal and alternative 3′ splice site to yield an exon-skipping product (Figure 5C and Figure S3A). These results suggested that in both natural evolution and artificial design, the positional order of two intron ribozymes with distinct splicing abilities is an important factor in controlling the selectivity of alternative splicing patterns. This mechanism may also occur in the exon-skipping splicing of two distinct classes of group I introns [26]. Exon-skipping with stepwise splicing was suppressed nearly completely by differentiating the primary sequence of the splice sites without disrupting their secondary structures and tertiary interactions.

While we propose rational strategies to restrict the splicing pattern of alternative splicing involving two group I introns, our strategies in this study were not practical for use in synthetic biology. Rational pairwise engineering of two introns and their splice site elements simply biases one of the two splicing patterns. Alternative splicing catalyzed by the spliceosome, however, is often regulated to selectively yield one of the multiple splicing products in a manner dependent on external factors [34,35]. For the practical application of ribozyme-based alternative splicing as a tool for gene regulation in RNA synthetic biology, the regulation of each intron ribozyme activity and splice site selection by external factors would be required. A straightforward direction for engineering the intron ribozyme is the installation of allosteric functions into each intron ribozyme. A naturally occurring allosteric group I ribozyme has been reported, in which the formation of the 5′ splice site was regulated by an aptamer module embedded in its P1 element [36]. The rational design of allosteric group I ribozymes has also been reported, in which the stability of the active ribozyme structures was controlled by the recognition of small molecules and RNA-binding peptides [37,38,39]. The introduction of these natural and rational strategies to the engineered introns developed in this study would expand the practical utility of ribozyme-based alternative splicing for use in RNA synthetic biology.

## 5. Conclusions

We constructed tandemly aligned *Tetrahymena* introns flanking a short exon that performed both exon-inclusion and exon-skipping splicing. The rational engineering of the two-intron ribozyme was performed in a pairwise manner to suppress one of two splicing patterns regarding their self-splicing in *E. coli*. Exon-skipping splicing was selectively conducted by pairs of mutant introns that formed a single large ribozyme. For selective exon-inclusion splicing, the suppression of exon-skipping splicing was achieved by engineering the core ribozyme structure and splice site recognition elements. These results will be useful for the development of ribozyme-based gene regulation systems for RNA synthetic biology.

## Data Availability

Not applicable.

## Supplementary Materials

The following supporting information can be downloaded at: https://www.mdpi.com/article/10.3390/biom13040654/s1, Figure S1: secondary structures of the *Tetrahymena* group I intron ribozyme and its engineered variants, Figure S2: splicing patterns of precursor RNAs possessing two *Tetrahymena* introns, Figure S3: splicing patterns of the tandemly aligned *Tetrahymena* group I introns and their rational design, Figure S4: exon-skipping splicing conducted by pairs of chimeric group I introns, Figure S5: rational engineering of tandemly aligned introns to induce exon-inclusion splicing, Figure S6: distal and alternative splice sites that induce exon-skipping splicing, Figure S7: rational engineering of 5′ and 3′ splice sites to distinguish two introns, Figure S8: suppression of exon-skipping splicing in tandemly aligned introns with distinct splice site recognition sequences.
