# Supplementary material for: Pairwise Engineering of Tandemly Aligned Self-Splicing Group I Introns for Analysis and Control of Their Alternative Splicing
Source: Biomolecules. 2023 Apr 6;13(4):654. doi: 10.3390/biom13040654 (PMC10136302; doi:10.3390/biom13040654)
Supplement: Supplementary file 1 [file biomolecules-13-00654-s001.zip › biomolecules-2291859-supplementary.pdf]

Supplementary Materials

For

Pairwise Engineering of Tandemly Aligned Self-Splicing Group I Introns for Analysis and Control of Their Alternative Splicing

Tomoki Ueda, Kei-ichiro Nishimura, Yuka Nishiyama, Yuto Tominaga,

Katsushi Miyazaki, Hiroyuki Furuta, Shigeyoshi Matsumura, Yoshiya Ikawa

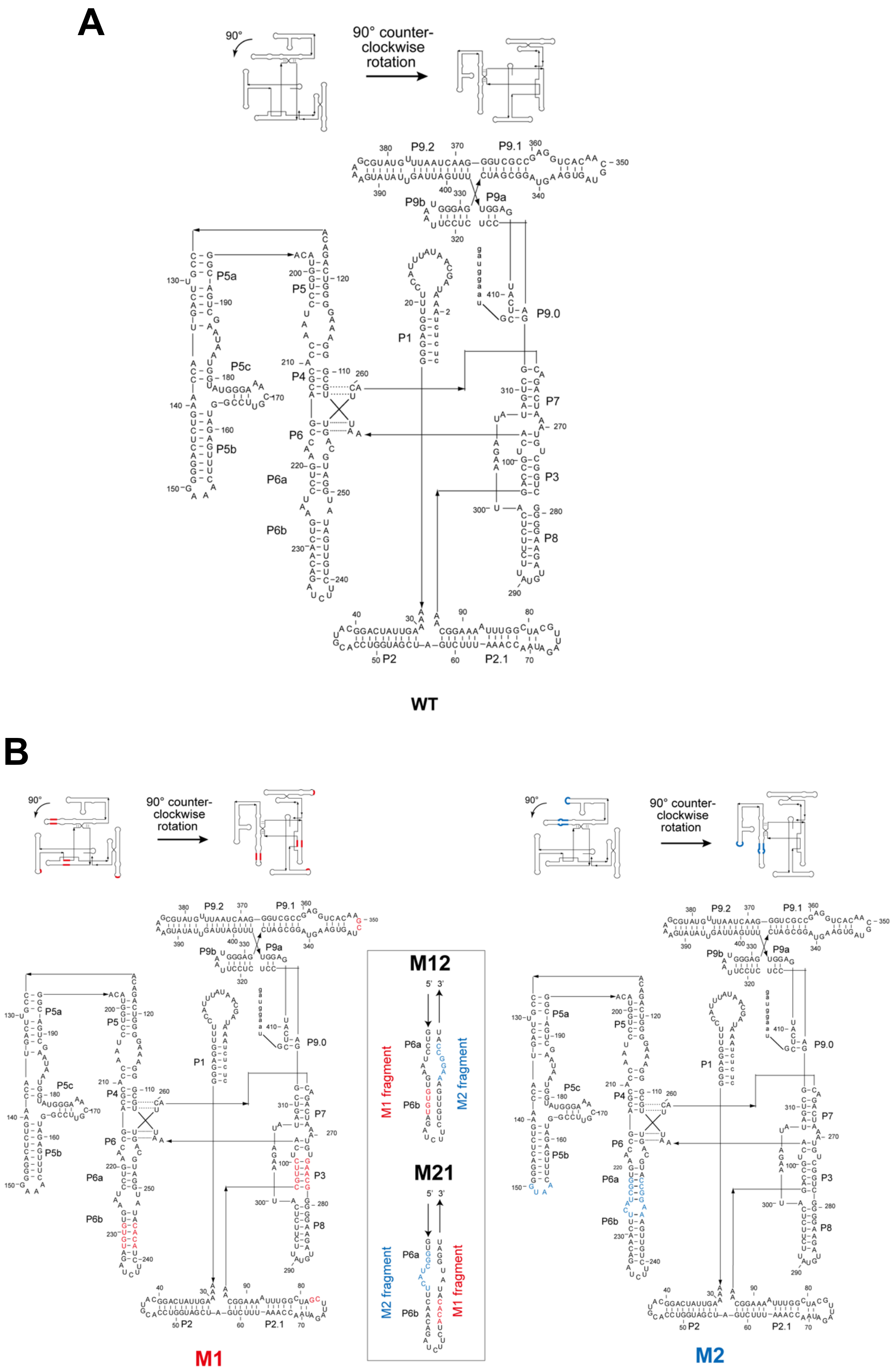
A
90°
90° counter-clockwise rotation
P9.2
P9.1
P9b
P9a
P9.0
P5a
P5
P5c
P4
P6
P5b
P6a
P6b
P1
P7
P3
P8
P2
P2.1
WT
B
M12
M21
5' 3'
M1 fragment
M2 fragment
M1
M2

Figure S1

C

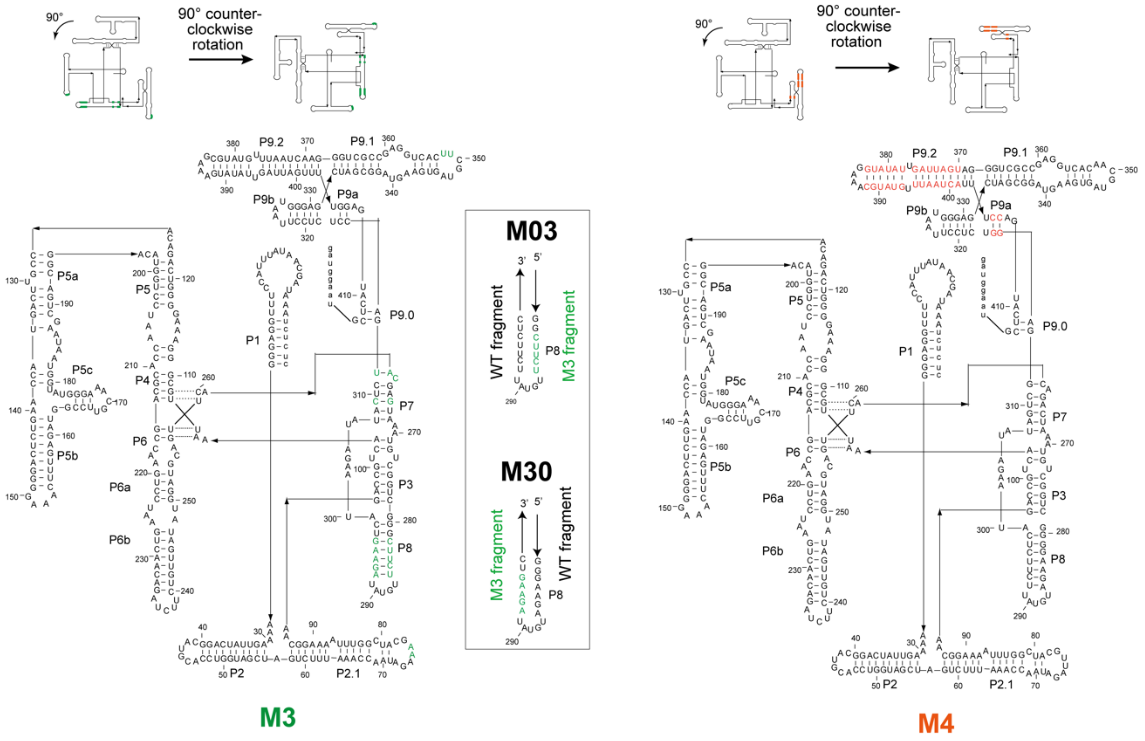

Figure S1

Secondary structures of the *Tetrahymena* group I intron ribozyme and its engineered variants

Shortened forms (L-21-ScaI forms) of M1-M4 variant RNAs catalyzed a reaction mimicking the first step of self-splicing (GTP-dependent cleavage of a substrate RNA), efficiency and accuracy of which were apparently comparable to those of the reaction catalyzed by the WT L21-ScaI RNA [28]. When bimolecular forms of the WT intron and M1-M3 variant RNAs were inserted into a fluorescent RNA aptamer (spinach aptamer), *in vitro* self-splicing (*trans*-splicing) of the four precursors proceeded in a cotranscriptional manner to produce the functionally active spinach aptamer [28].

A) Secondary structure of WT *Tetrahymena* group I intron.

B) Secondary structures of M1 and M2 variants. Nucleotides colored red in M1 and blue in M2 indicate sequences different from those of the WT intron. P6 elements of chimeric introns M12 and M21 are also shown between M1 and M2 variants.

C) Secondary structures of M3 and M4 variants. Nucleotides colored green in M3 and orange in M4 indicate sequences different from those of the WT intron. P8 elements of chimeric introns M03 and M30 are also shown at the left of the M3 variant.

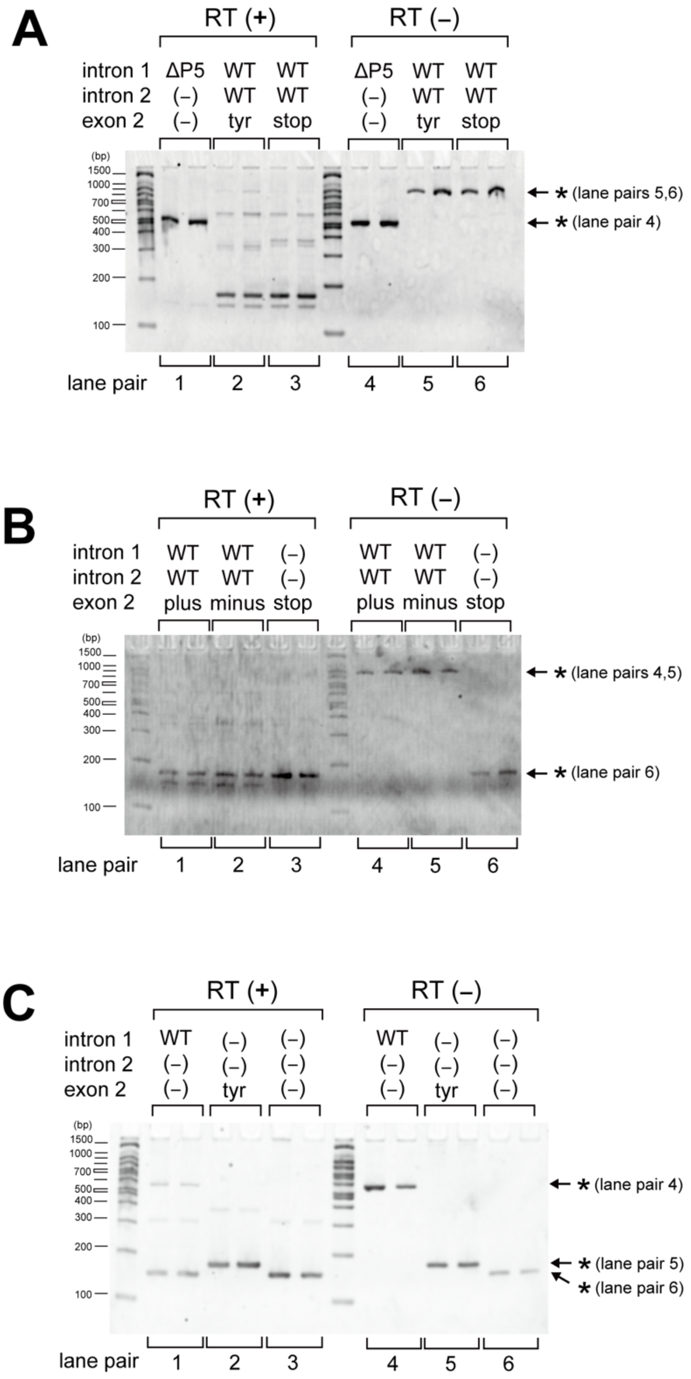

Figure S2

Splicing patterns of precursor RNAs possessing two *Tetrahymena* introns

A–C) RT-PCR assays to distinguish the ligated exons (E123 or E13) including or skipping exon 2, with lengths of 174 and 150 bp, respectively. As the ΔP5 mutant was splicing-deficient in *E. coli*, it provided the RT-PCR product corresponding to the precursor RNA. In lane pairs 1–3 and 4–6, PCR was performed with or without reverse transcription (RT), respectively. Asterisks indicate PCR products amplified from the residual plasmid DNA in the absence of RT.

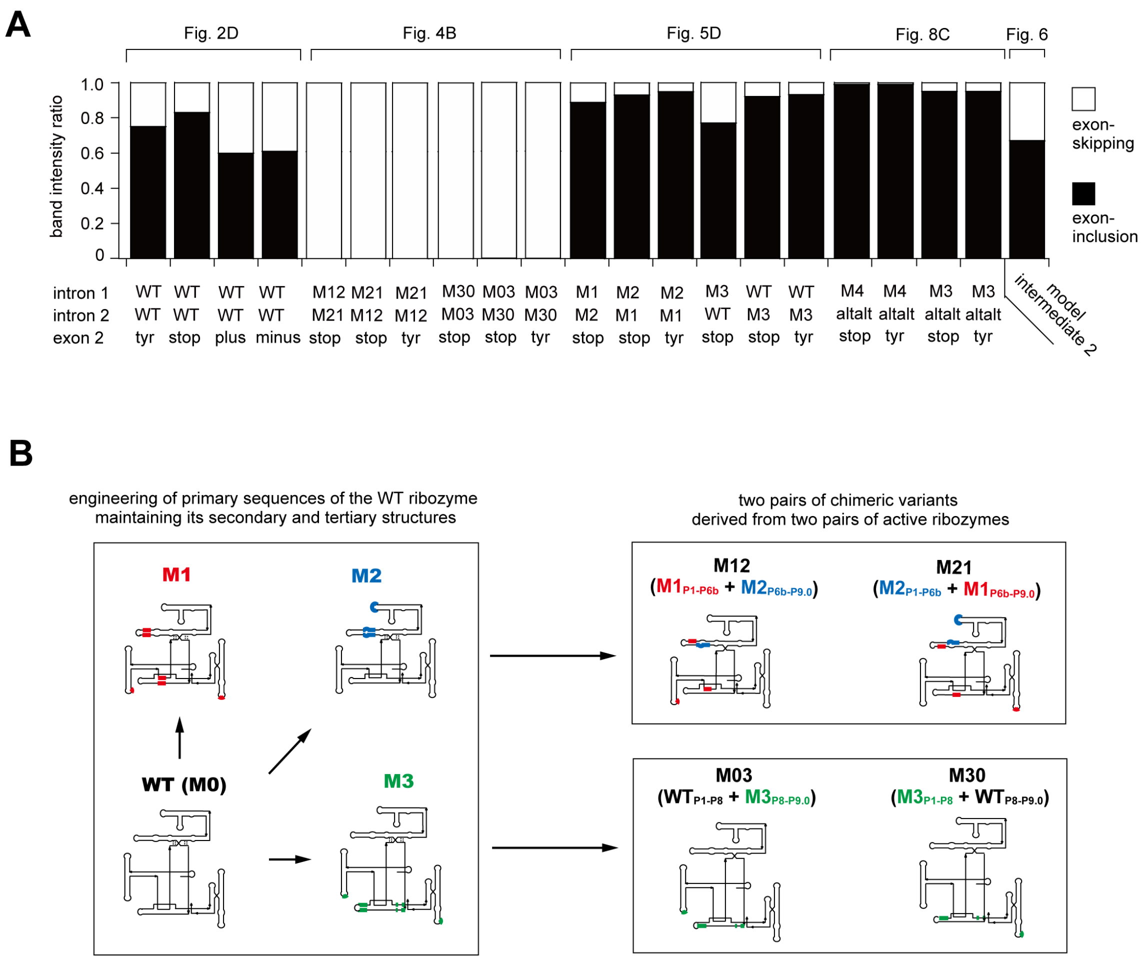

Figure S3

Splicing patterns of the tandemly aligned *Tetrahymena* group I introns and their rational design

A) Relative amounts of RT-PCR products corresponding to exon-inclusion splicing (black) and exon-skipping splicing (white).

B) Engineering of the WT *Tetrahymena* intron to generate M1–M3 variants (left) and further engineering to generate two pairs of chimeric variants.

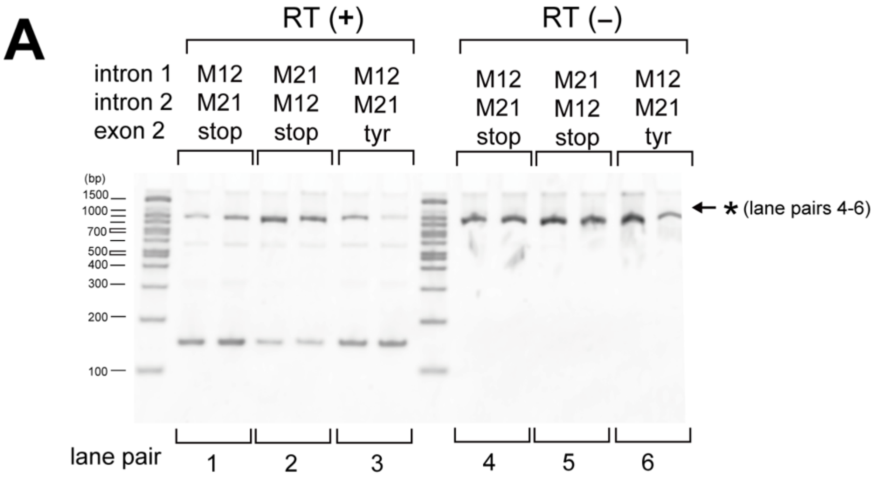

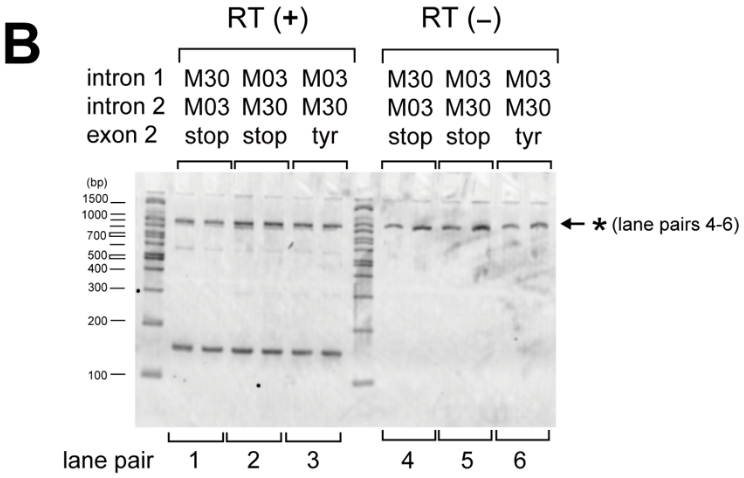

Figure S4

Exon-skipping splicing conducted by pairs of chimeric group I introns

A, B) RT-PCR assays to distinguish the ligated exons (E123 or E13) including or skipping exon 2, with lengths of 174 and 150 bp, respectively. In lane pairs 1–3 and 4–6, PCR was performed with or without reverse transcription (RT), respectively. Asterisks indicate PCR products amplified from the residual plasmid DNA in the absence of RT.

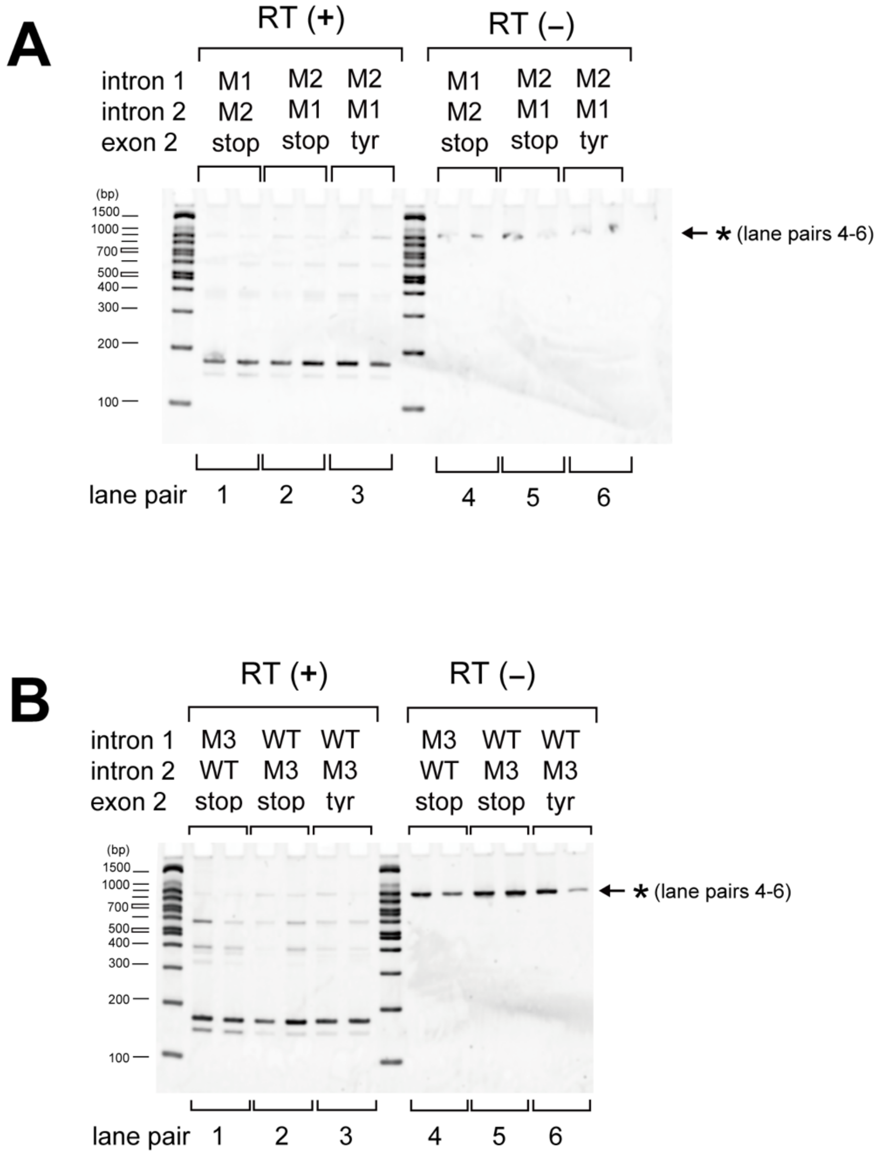

Figure S5

Rational engineering of tandemly aligned introns to induce exon-inclusion splicing

A, B) RT-PCR assays to distinguish the ligated exons (E123 or E13) including or skipping exon 2, with lengths of 174 and 150 bp, respectively. In lane pairs 1–3 and 4–6, PCR was performed with or without reverse transcription (RT), respectively. Asterisks indicate PCR products amplified from the residual plasmid DNA in the absence of RT.

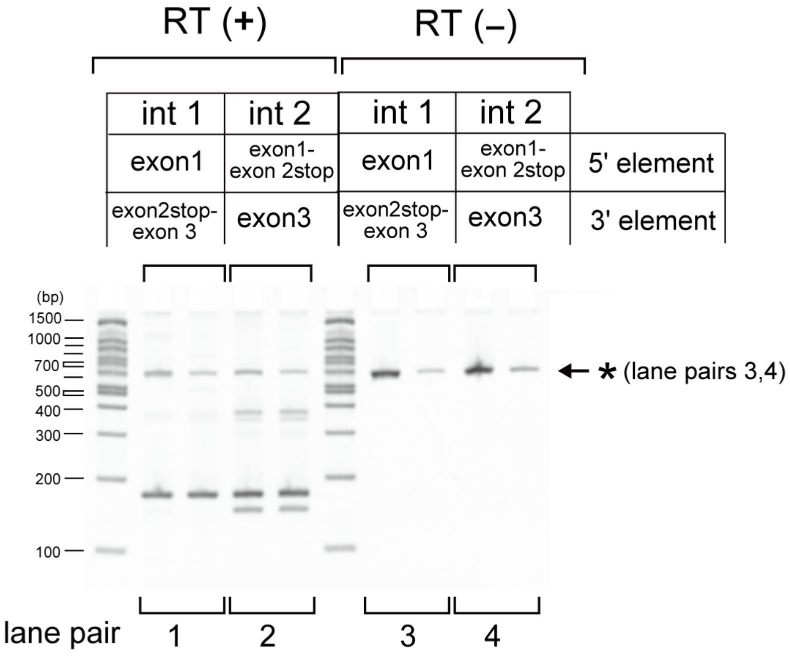

Figure S6

Distal and alternative splice sites that induce exon-skipping splicing

RT-PCR assays to distinguish the ligated exons (E123 or E13) including or skipping exon 2, with lengths of 174 and 150 bp, respectively. In lane pairs 1–2 and 3–4, PCR was performed with or without reverse transcription (RT), respectively. Asterisk indicates PCR product amplified from the residual plasmid DNA in the absence of RT.

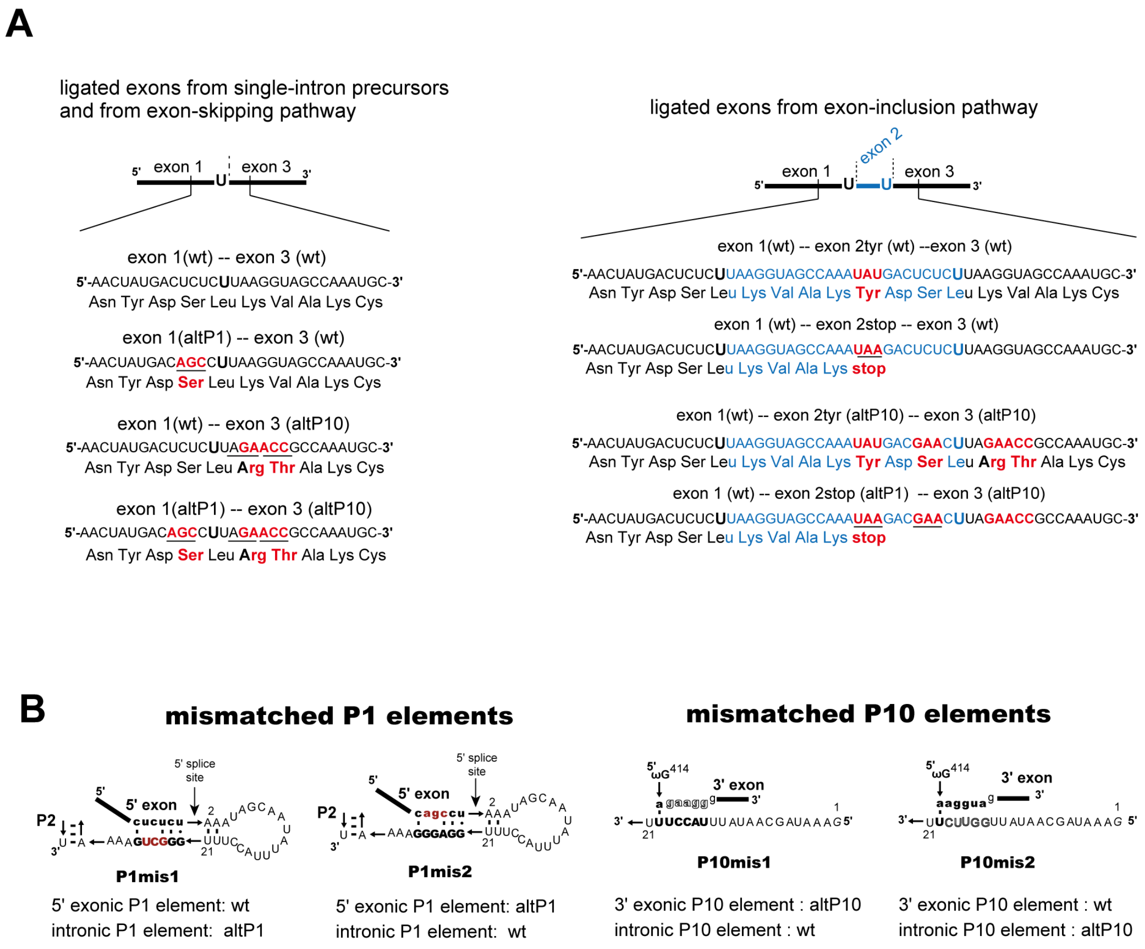

Figure S7

Rational engineering of 5′ and 3′ splice sites to distinguish two introns

A) Nucleotide sequences of the ligated exons from variant 5′ and/or 3′ elements.

B) Mismatched P1 elements (left) and P10 elements (right) that inhibit and reduce the production of the ligated exons.

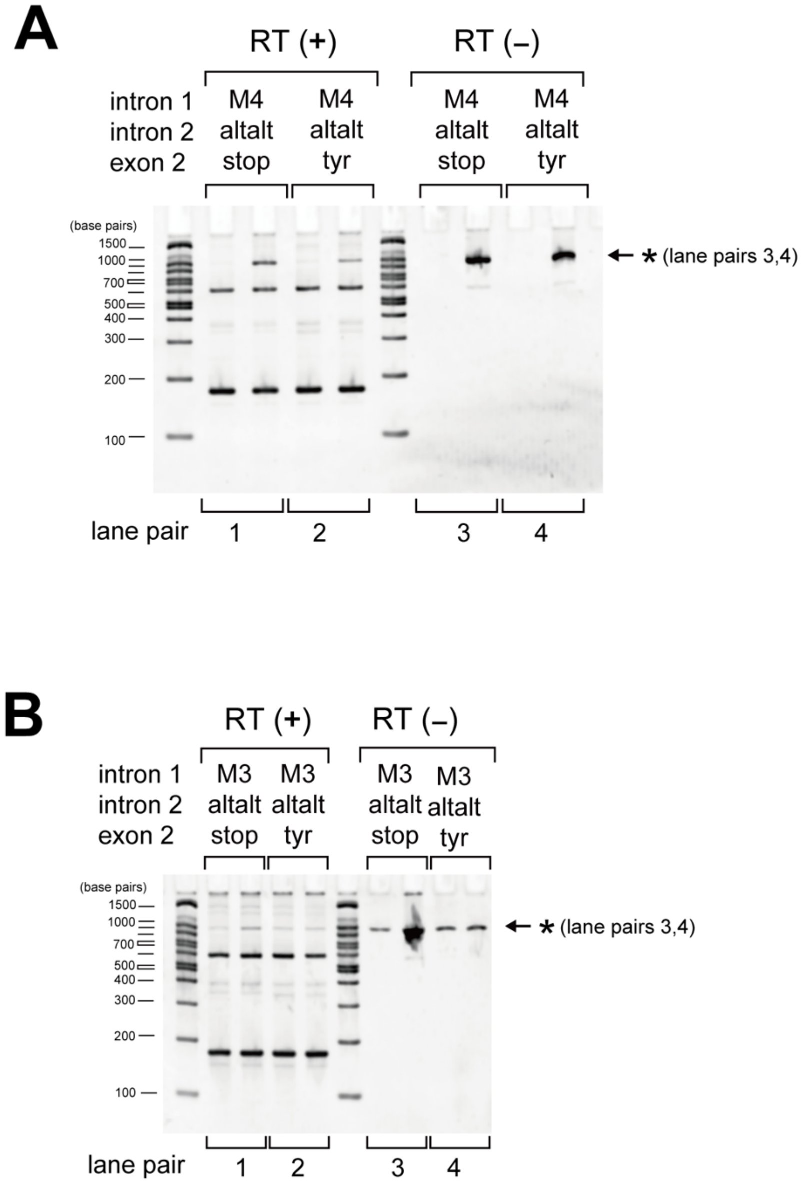

Figure S8

Suppression of exon-skipping splicing in tandemly aligned introns with distinct splice site recognition sequences

A, B) RT-PCR assays to distinguish the ligated exons (E123 or E13) including or skipping exon 2, with lengths of 174 and 150 bp, respectively. In lane pairs 1–2 and 3–4, PCR was performed with or without reverse transcription (RT), respectively. Asterisks indicate PCR products amplified from the residual plasmid DNA in the absence of RT.
